# Activity in Barrel Cortex Related to Trace Eyeblink Conditioning

**DOI:** 10.1523/ENEURO.0206-23.2023

**Published:** 2023-08-21

**Authors:** May-Li Silva-Prieto, Julian I. Hofmann, Cornelius Schwarz

**Affiliations:** Werner Reichardt Center for Integrative Neuroscience, Hertie Institute for Clinical Brain Research, Systems Neurophysiology, Eberhard Karls University, Tübingen, Germany

**Keywords:** generalization, memory systems, mouse, pavlovian conditioning, spike plasticity, trace conditioning

## Abstract

In mammals several memory systems are responsible for learning and storage of associative memory. Even apparently simple behavioral tasks, like pavlovian conditioning, have been suggested to engage, for instance, implicit and explicit memory processes. Here, we used single-whisker tactile trace eyeblink conditioning (TTEBC) to investigate learning and its neuronal bases in the mouse barrel column, the primary neocortical tactile representation of one whisker. Behavioral analysis showed that conditioned responses (CRs) are spatially highly restricted; they generalize from the principal whisker only to its direct neighbors. Within the respective neural representation, the principal column and its direct neighbors, spike activity showed a learning-related spike rate suppression starting during the late phase of conditioning stimulus (CS) presentation that was sustained throughout the stimulus-free trace period (Trace). Trial-by-trial analysis showed that learning-related activity was independent from the generation of eyelid movements within a trial, and set in around the steepest part of the learning curve. Optogenetic silencing of responses and their learning-related changes during CS and Trace epochs blocked CR acquisition but not its recall after learning. Silencing during the Trace alone, which carried major parts of the learning-related changes, had no effect. In summary, we demonstrate specific barrel column spike rate plasticity during TTEBC that can be partially decoupled from the CR, the learned eye closure, a hallmark of implicit learning. Our results, thus, point to a possible role of the barrel column in contributing to other kinds of memory as well.

## Significance Statement

Association learning relies on different memory systems. The pavlovian eyeblink reflex conditioning paradigm already involves the generation of a new movement (move the eyelid, implicit memory), and to learn the task contingencies (know the rule of the game, explicit memory). Using this paradigm, we study the involvement of whisker-related mouse primary somatosensory cortex (S1) in different memory systems. With recording and causal stimulation techniques, we found spike plasticity in S1 during presentation of the conditioned stimulus that is causal for implicit learning. Notably, however, blocking learning-related changes during the stimulus-free trace period did not impair implicit memory. We conclude that S1 activity is related to the implicit and at least one other memory system, presumably the explicit one.

## Introduction

Associations help us learn the temporal and/or spatial correlation between behaviorally relevant events, be they events in the world, our own perceptions, actions, or behavioral dispositions. In mammals, several behaviorally defined memory systems work in parallel and in distributed neuronal networks to store an association (Schacter and Tulving, 1994). The task to map memory systems to neuronal structure and behavior therefore faces the often-ignored difficulties that multiple behavioral memory systems are engaged even by simple learning tasks, that they are widely distributed in the brain, and that they eventually even may engage overlapping neuronal structures. A prime example is the simple learning task called trace eyeblink conditioning (TEBC), a form of pavlovian conditioning, where the conditioned stimulus (CS; a neutral sensory stimulus) and unconditioned stimulus (US; a corneal stimulus reflexively evoking eye closure) are presented with a gap in between. This gap (called Trace period here) needs to be bridged by a memory trace (hence the name) to achieve the association. It is well established that TEBC requires the participation of hippocampus and neocortex in addition to the well-established contribution of the cerebellum (Hesslow and Yeo, 2002; Woodruff-Pak and Disterhoft, 2008; Kalmbach et al., 2009). Traditionally, the required cerebral trace signal has been implicated to inform the implicit memory system, namely, to carry the trace signal through the gap to be able to learn the generation of the conditioned response (CR) in response to the CS. However, it is common wisdom that the cerebral structures in question certainly are able to generate other types of memory. For example, declarative memory in humans is based on the cerebral cortex and generates verbally reportable knowledge about task contingencies. It is by now a classical result that during eyeblink conditioning declarative memory is generated parallel to the implicit type in humans, and the two types of memory are readily discernible by studying their characteristic content, namely CRs for the implicit versus vocal report for the explicit system (Clark and Squire, 1998; Clark et al., 2001).

Here, we studied an animal model of TEBC, the tactile TEBC (TTEBC), in the whisker system in mice. The primary somatosensory barrel cortex (whisker representation of the primary somatosensory cortex in rodents) is known to play a distinct role in TTEBC as evidenced by the requirement of its intactness (Galvez et al., 2007), the presence of spatially specific map plasticity (Galvez et al., 2006; shown in rabbits), and the reshaping of micro networks in the barrel column (Ward et al., 2012; Chau et al., 2014; Joachimsthaler et al., 2015). Further, it offers many advantages such as its well-defined anatomic boundaries and accessibility for neuronal recordings (Woolsey and Van der Loos, 1970; Van Der Loos, 1976; Ma and Woolsey, 1984), as well as its embedment within a rich set of sensorimotor and cognitive neural networks (Bosman et al., 2011; Feldmeyer et al., 2013; Stüttgen and Schwarz, 2018).

As verbal reports in the animal model are difficult to come by, we did not attempt to study explicit memory directly. Instead, we used novel systematic trial-by-trial analyses of learning behavior and multielectrode extracellular recordings to explore when and how known neocortical players contribute or not to the learning of implicit content. Confirming previous results about map and spine plasticity (Galvez et al., 2006; Joachimsthaler et al., 2015), we established the presence and causal role of spatially very specific spike plasticity during the presentation of the CS as well as during the Trace. Employing optogenetic epoch-specific blockade of barrel cortex, we found that plastic neuronal signatures during CS contribute to generate implicit memory (CRs), whereas those observed during Trace did not. From these results we speculate that Trace plasticity in barrel cortex serves another type of memory, perhaps that of the explicit type.

## Materials and Methods

### Animals

Mice were treated in full compliance with the German Law for the Protection of Animals (licensed by Regierungspräsidium Tübingen). Adult, male wild-type *(C57BL/6N)* and VGAT (vesicular GABA transporter; *VGAT-ChR2-EYFP*; Zhao et al., 2011) mouse lines were used. The animals were housed on an inverted 12/12 h light/dark cycle with food and water *ad libitum*.

### Surgeries

The implantation surgeries were performed under three-component fentanyl anesthesia, containing fentanyl, midazolam, and meditomidine. Anesthesia was induced by intraperitoneal injection of 0.05 mg/kg fentanyl (Ratiopharm), 5 mg/kg midazolam (hameln pharma), and 0.5 mg/kg medetomidine (Sedator, Eurovet Animal Health) and maintained by one-third of the initial dose, administered every 1–2 h. Throughout the surgery, eyes were covered by a moisturizing ointment (Bepanthen, Bayer). Body temperature was measured by a rectal probe and maintained at 37°C using a homeothermic pad. After shaving and disinfection, the skin was incised and the pericranium retracted. The skull was cleaned using 3% hydrogen peroxide solution and coated with a light curing bond (OptiBond FL; Kerr) and a thin layer of dental cement (2 mm frontal and 4 mm lateral; Tetric EvoFlow, Ivoclar Vivadent), sparing the trepanation site. A trepanation of ∼2 × 2 mm was drilled, −2 mm frontal and 4 mm lateral to bregma on the right hemisphere above the barrel cortex, leaving the dura mater intact. To identify the barrel map, intrinsic optical imaging was performed on the E1 and surrounding whiskers. E1 barrel columns were implanted with electrodes (wild-type mice, see below) or an optical fiber (VGAT mice, see below). In case of electrode implantation, two silver ball electrodes were placed on the surface of the cerebellum, serving as ground and recording reference. A 10 mm M3 screw was embedded into the head cap head down for head fixation. At the end of surgery, the anesthetic agents were antagonized, using a subcutaneous injection of 1.2 mg/kg naloxone (hameln pharma), 0.5 mg/kg flumazenile (Frisenius Kabi), and 2.5 mg/kg Atipam (Eurovet Animal Health). As postsurgical treatment the animals received 3 d of analgesic medication (5 mg/kg, s.c., twice per day of carprofen; Rimadyl, Zoetis) and 7 d of antibiotic medication (oral dose, Baytril, Bayer).

### Intrinsic optical imaging

Intrinsic imaging was used to functionally map the location of the CS-activated barrel column exactly as described in Joachimsthaler et al. (2015). Briefly, the surface blood vessel pattern was captured using green light (570 nm) as reference. The intrinsic optical signal was captured by monochromatic red light (630 nm) with a charge-coupled device camera focused on a cortical depth of 200–250 μm while a single whisker was deflected using a 60 Hz sine wave, rostrocaudal amplitude 0.7 mm at 5 mm distance from the face. The activated area by single-whisker deflection was automatically traced by boxcar filtering the image (kernel 10 × 10 pixels), followed by normalization of the range of captured gray values to [0, 255] and adjusting a threshold of gray values to capture an activation area approximately the diameter of a barrel (300 μm).

### Electrophysiology

Seven wild-type C57BL/6N mice were chronically implanted either (in six cases) with 32-channel, 4-shank silicone probes or in one mouse with a 16-channel, 1-shank silicone probe (catalog #E32-150-S4-L2-200, catalog #E16+R-100-S1-L6 NT, Atlas Neuroengineering) to record extracellular neuronal activity during TTEBC acquisition. The shanks of these probes measured 80 × 50 µm in diameter, had their tips sharpened, and carried low-impedance platinum electrodes with a diameter of 35 µm. In the four-shank probe the electrode distance along the shank was 200 µm (total range, 600 µm), and a spacing of 150 µm between shanks (total range, 1050 µm). The one-shank probe had 16 electrodes separated by 100 µm. The silicone probes were implanted in barrel cortex spanning all cortical layers. On the surface of barrel cortex, the silicon probe was oriented so that the first shank entered the E1 barrel column, and the fourth shank roughly sat in D1/C1, thus covering the principal column and at least one adjacent one.

Recordings were made against a silver ball electrode implanted on the surface of the cerebellum. The possibility that bulk signals picked up from the cerebellum influenced our results can be safely excluded. First, we observed learning-related responses in spike data; they can be excluded to originate from eventual learning-related bulk signals picked up by the silver ball reference. Second, we found a clear spatial profile of neuronal responses both in depth and along horizontal distance in the local field potential (LFP) as well as spike data. Third, from each of the spike recordings we subtracted the mean obtained across all electrodes, a measure that significantly reduces any global influences (e.g. ambient noise, animal movement, or reference signals).

### Optical fiber implants and optogenetic experiments

*VGAT-ChR2-EYFP* mice (Zhao et al., 2011; Guo et al., 2014) were chronically implanted with fiber implants to shine light on the surface of the barrel cortex (BCx) during TTEBC. The implant was assembled from a short piece of 400 µm light fiber (0.39 NA, FT400UMT; Thorlabs) and a custom-made alloy ferrule (either new silver or bronze) with an outer diameter of 2.5 mm and a length of ∼7 mm. The fiber was glued into the ferrule with epoxy resin so that the side pointing toward the brain projected out by ∼3 mm. Both sides of the fiber implant were grinded thoroughly to maximize light input and output. The fiber implant was placed and fastened directly on the cortical surface, straight above the E1 barrel.

In preliminary experiments the effectiveness of optogenetic BCx perturbation was tested in anesthetized mice. The surgical methods and anesthesia were the same as described above for the implantations. After the trepanation, a high-impedance (>3MΩ) glass electrode was lowered into BCx, recording single units at various cortical depths. A 400 µm light fiber was placed on the cortical surface, above the spot of the electrode position, through which a constant 4.3 mW light pulse of 500 ms duration was applied 50 times at 0.2 Hz.

In optogenetic experiments with fiber-implanted animals a 470 nm blue high-power LED (32; catalog #NCSB119, Nichia) mounted on a custom-made aluminum holder served as the light source for the optogenetic experiments. The light was fed into an 800 µm fiber (0.39 NA, catalog #FT800UMT; Thorlabs) which was in turn coupled to the 400 µm fiber implant by a 2.5 mm, ceramic mating sleeve, creating a very tight, stable, and reproducible fiber connection. The LED was controlled by an LED driver (LEDD1B; Thorlabs) generating a continuous light pulse of 4.3 mW output intensity at the cortex surface. This intensity was at least threefold higher than the one needed to efficiently suppress excitatory spiking throughout all cortical layers in awake mice. To minimize rebound firing at light offset, each light pulse was dimmed on a linear decay trajectory and reached zero intensity after 500 ms (Guo et al., 2014).

### Behavioral experiments

The animals were studied using head fixation, providing highest stimulus and experimental control (Schwarz et al., 2010). The animals were thoroughly handled and habituated to head fixation in several daily sessions for at least 2 weeks before data acquisition. Data acquisition, consisting of behavioral assessment of learning and recording of multielectrode extracellular signals, was performed for a minimum of five sessions on 5 consecutive days, always at the same time of the day.

**Figure 1.**
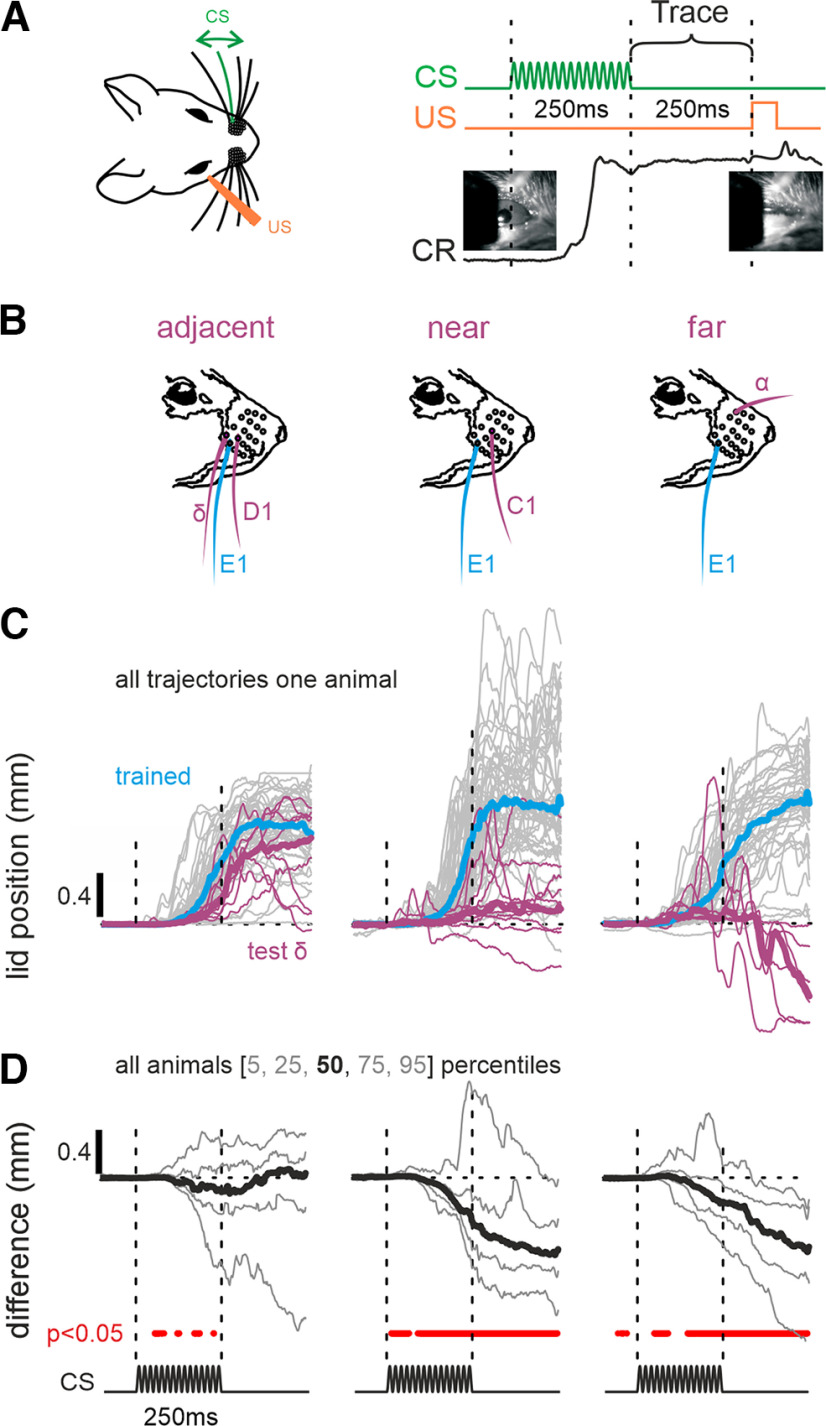
Generalization of TTEBC. ***A***, Left, Experimental setup. Head-fixed mice received sinusoidal movement of a single whisker (60 Hz, amplitude 5°, duration 250 ms) as a CS, paired with an air puff against the cornea as a US. Right, CS and US presentations were separated by a 250 ms stimulus-free period (tagged Trace). The mice learned to associate the CS and US as read out by the generation of a CR, an eyeblink. The CR response (lid position, black line, the lid movement is ∼1 mm in the depicted case) started already during the CS in all mice studied. ***B***, Schematic of whiskers used to study the generalization of the learned response (the whisker used to train TTEBC, blue; the test whiskers, magenta). ***C***, Eyelid trajectories as measured during presentation of the trained whisker (blue) and test whisker (nontrained, magenta). After acquisition of the task, several sessions were performed in each of which 50 E1-US and 10 test stimuli (without US) were presented. Thin lines, eyelid trajectories of one representative mouse. Thick lines, median trajectory. ***D***, Difference between eyelid movements observed with stimulation of the test whisker and median of the trained whisker (data from 4/3/4 mice for adjacent/near/far test stimuli). Percentile trajectories 5, 25, 50, 75, 95 are depicted (median, thick line; the others, thin lines). Left, The red dots indicate a significant difference obtained using the sign test (*n* = 34/28/36, adjacent/near/far), *p* < 0.05).

TTECB conditioning was conducted as reported previously (Fig. 1*A*; Joachimsthaler et al., 2015). Briefly, one session contained the presentation of the exact same number of stimuli per session (60 CS and 60 US per session). TTEBC presented CS and US in a paired fashion with a fixed 250 ms stimulus-free (Trace) period intercalated between CS and US. The pairs were separated by random picks from a flat distribution of intertrial intervals between 20 and 40 s. The CS was a 60 Hz sinusoidal whisker deflection for 250 ms (15 periods) at a position amplitude of 5° and a velocity amplitude of 1870°/s. The US was a corneal air puff from a distance of 3 mm via a thin 200 μl pipette at 40 psi, a strength that securely leads to a reflexive eye closure (unconditioned response) when presented alone. To mask any possible acoustic emission by the tactile stimulator, a 60 dB white noise was present at all time. Eyeblinks were recorded at 2 kHz throughout the training session, using an infrared (IR) light source and sensor (OPR5005, Optek), placed in proximity to the eye, to which the US was applied (Weiss and Disterhoft, 2008). Any eye closure affects the amount of reflected IR light, causing a voltage change on the sensor (Joachimsthaler et al., 2015).

The behavioral paradigm to test generalization (Fig. 1) first trained the animals on TTEBC (five sessions, 300 trials), using deflection of whisker E1. Then postlearning sessions were run in which two actuators were attached, one to whisker E1 and one to another the test whisker. Among test whiskers, D1 or δ whisker (adjacent), C1 whisker (near), and α whisker (far) were used. The whiskers chosen were from the Greek arc or arc 1, all among the longest rodent macro vibrissae with comparable biomechanical and neuronal sensitivities (Hartmann et al., 2003; Neimark et al., 2003). Combined with driving them in the far suprathreshold regime (60 Hz position amplitude 5°; velocity amplitude 1870°/s; cf. Stüttgen et al., 2006), significant detection/response differences are likely negligible and are highly unlikely to affect the measurement of any significant gradient of generalization. In these generalization sessions, one randomly selected trial within every block of six trials deflected the test whisker instead of the trained E1 (deflection parameters identical). The test stimulus was never paired with a US to avoid new conditioning. Each whisker was tested in one session, containing 50 training trials and 10 test trials. The tests were performed in sequential blocks from far via near to adjacent whiskers. To compare eyelid movements across sessions, the pre-CS eye position was subtracted from all traces, and the trajectories were pooled for adjacent, near, and far whiskers.

### Classification of CR versus non-CR

We used a one-dimensional convolutional neural network (CNN; TensorFlow version 2.0, Python software) to classify CRs versus non-CRs (nCRs). The CNN consisted of three convolutional layers and three neural layers with dropout regularization to avoid overfitting. This classifier used 2109 eye traces from previous behavioral trainings, which were manually labeled in three categories, CRs, non-CRs, and closed eyes. The CR category consisted of eye traces that had a sharp voltage increase during CS presentation, indicating a partial eye closure of minimally 20% of that seen after the US and was upheld or increased during the remaining trial time (through CS and Trace periods) until the US was presented. Failure to reach these criteria meant that the eye traces were either classified as closed eye, where the baseline pre-CS voltage was similar to that of the US, or a non-CR, where none of the previous criteria applied. We used 85% of the eye traces for training (*n* = 1793) and excluded 15% to use as validation dataset (*n* = 316). After training, the accuracy of the model was 94.2%, as estimated by classifying the validation dataset. All CNN classifications were double-checked by eye using semiautomatic, custom-made software (MATLAB).

### Constructing single animal/trial-resolved learning curves

**Figure 2.**
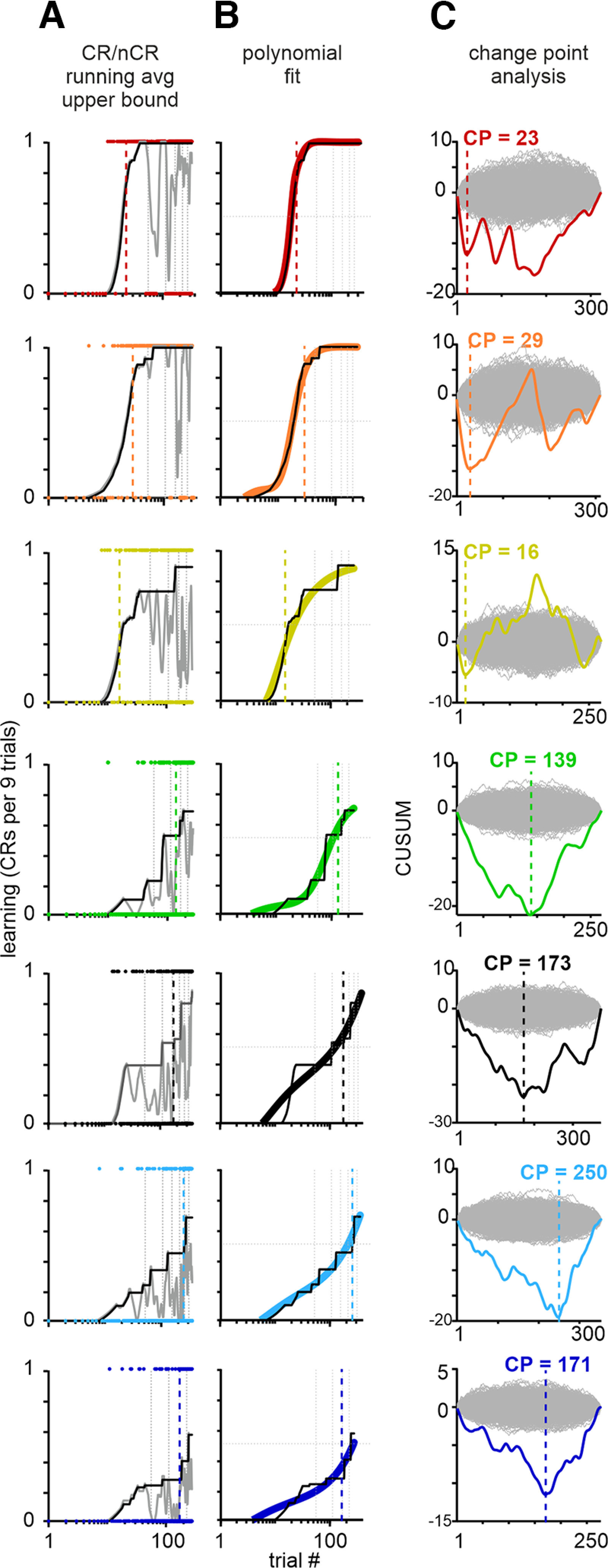
Learning curves. ***A***, Raw learning data (binary data of trials containing conditioned and no conditioned responses; CR = 1, nCR = 0) in the seven trained mice (indicated by colors) implanted with electrode arrays in the barrel cortex (colored dots). Gray lines depict a running average of the binary data (kernel width 9 trials). Assuming learning progress is described by a monotonically increasing curve, the black line is an upper bound of learning generated by following the running average when it is increasing and staying constant if it is decreasing. ***B***, A polynomial fit (curves in colors) to the upper bound learning curve (black curves). The fits are used in Figure 6. ***C***, Change point analysis. The colored curves depict the CUSUM curve, which is the cumulative difference between a series of data points and their mean. Gray lines are a bootstrapped population of 1000 CUSUM curves generated from randomly shuffled time series. Peaks in the CUSUM curve indicate potential change points (negative, a change to a higher mean; positive, a change to a lower mean). In the present context of learning data only negative peaks are relevant. We selected the first negative peak between the point of departure from the bootstrapped data and its reentry as the change point for learning behavior (vertical broken lines). The trials at which change points (CP) were found are indicated and are used in Figure 5. The change points have been added to ***A*** and ***B*** as well to facilitate comparison with the smooth learning curve. Note the logarithmic scale abscissae in ***A*** and ***B*** versus the linear one in ***C***.

After classifying trials, the raw learning process of each animal was reflected by a binary vector with a number of bins equal to the number trials [no conditioned response (nCR) = 0, CR = 1; dots in Fig. 2*A*]. As explained in more detail later (see below, Results), we assumed that the dynamics of a trial-resolved learning score in an individual animal can show specific deviations from simple S-shaped curves classically used to fit to them (Gallistel et al., 2004). Taking these notions into account, we quantified learning performance by independently calculating two variables. The first approach dropped the assumption of the parametric S shape but keeping the idea that learning develops in monotonic fashion (i.e., forgetting in the studied interval is negligible). It first estimates an upper bound to the learning curve by taking the value of the running average if its slope at the current trial was positive but keeping the last value (current trial minus 1) if the slope was negative (Fig. 2*A*,*B*, black curves). This resulted in a monotonic curve with sudden steps. A smooth progression of the learning score was estimated by fitting the upper bound with a higher-order polynomial (Fig. 2*B*, curves in colors). The second approach dropped all assumptions about gradation of learning and focused solely on so-called change point of learning, defined as the latency at which the animals first starts to respond (Gallistel et al., 2004). To this end we calculated the cumulative sum chart (CUSUM) curve, which is the cumulative difference between a series of data points and their mean [ $CUSUM\left(i\right)=\underset{j\leq i}{\sum }(S_{j}-\bar{S});$ with S the time series, i, j indices of time series, and $\bar{S}$ the mean of the time series; Fig. 2*C*; http://www.variation.com/cpa/tech/changepoint.html; Durstewitz et al., 2010; Chakrabarti et al., 2022]. The negative peaks of CUSUM curves (Fig. 2*C*, colors) indicate upward change points. The first peak outside the field of curves constructed from a bootstrapped population (Fig. 2*C*, gray lines) was taken as the change point of the behavior of the mouse (Fig. 2*C*, vertical dashed line).

### LFP/current source density analysis

**Figure 3.**
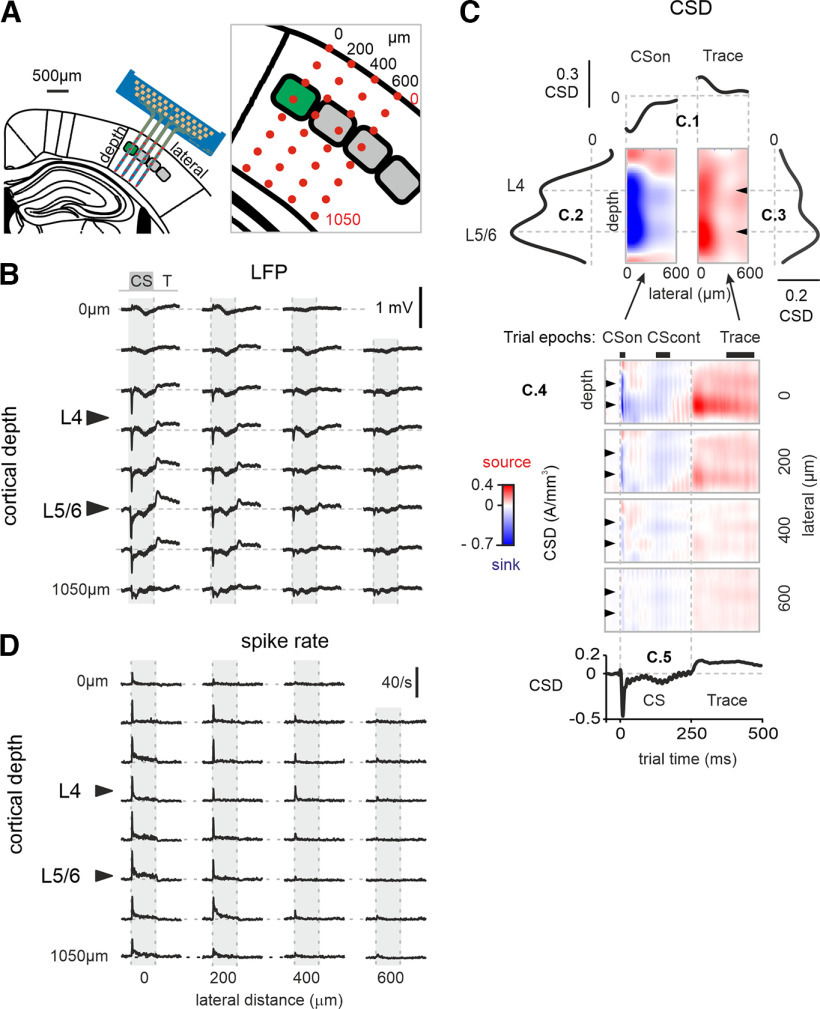
Electrophysiological recordings. Representative example. Averages from the first session recorded in the yellow mouse. ***A***, Schematic of recording via a 8 × 4 silicon-based electrode array (blue). Left, Frontal section of one hemisphere of a mouse brain. Right, Blowup of electrode locations (size of individual electrodes exaggerated for visualization in red). Based on intrinsic imaging of barrel columns, the shanks were implanted to span barrels E1 (principal), D1, and perhaps C1. Barrels in L4 of the barrel cortex are shown in the schematic (principal whisker, green). ***B***, Trial-averaged LFP responses obtained from a 8 × 4 electrode silicon probe in the first learning session (rows, depth; columns, lateral cortical distance). Trial epochs are marked on top of one column (T, Trace; trial time from −50 to 500 ms; CS is marked in gray). Vertical dashed lines depict onset and offset of CS. Horizontal dashed lines are zero potential. Black triangles mark L4 and L5/6 as determined from the CSD analysis (***C***). Note the sharp negative response to CS onset, most prominent between L4 and L5/6. It reverses in upper layers and decays along lateral distance (shanks). ***C***, Two-dimensional CSD analysis. Dimensions shown are trial time, cortical depth (electrodes), lateral distance (shanks), and CSD (color, units A/mm^3^; color scale blue, current sink; red, current source). CSD across cortical surface (***C.1***); CSD across layers (***C.2***, ***C.3***); two-dimensional CSD panes across depth and trial time (***C.4***); CSD across trial time averaged across depth between L4 and L5/6 (***C.5***). Analyzed trial epochs are shown above ***C.4***. CSon, onset response to CS; CScont, adapted response to CS; Trace, late in the gap between CS and US. Intervals (in ms), CSon, 5–25; CScont, 125–175; Trace, 375–475 ms (times relative to CS onset). ***D***, Multiunit spike rates. Note the sharp excitatory response at CS onset with a maximum between L4 and L5/6. It decays markedly across lateral cortical distance (shanks). Conventions are as in ***B***.

The LFP signal was derived from the raw recordings by downsampling to 2 kHz, and low-pass filtering (Butterworth filter, edge frequencies 1 and 200 Hz; filter passband ripple amplitudes < 0.5 dB, stopband attenuation > 30 dB). The matrix of LFP signals was converted into a current source density (CSD) map (Nicholson and Freeman, 1975; Mitzdorf, 1985) using the CSD reports where and when currents enter or exit the spanned extracellular space. We call a negative CSD a “sink” (blue in CSD maps) because of positively charged ions entering a cellular compartment (exit the extracellular space) or negatively charged ions leaving the cell (entering the extracellular space). The opposite, a positive CSD, is called “source” (colored in red). We used the kernel-based current source density method (Potworowski et al., 2012), which estimates the two dimensional CSD space spanned by the silicon shanks. Adding binned trial time and session numbers, we ended up having a 4D matrix of CSD values. This 4D matrix was then averaged and cropped to arrive at matrices of lower dimensionality (Fig. 3).

### Spike analysis

The average potential obtained across the entire electrode array (32 channels, in one case 16 channels) was subtracted from each channel to reject common noise sources (e.g., potentials at the silver ball electrode and movement artifacts, etc.). Hereafter, the data were bandpass filtered (Butterworth filter, edge frequencies 500 and 3000 Hz; filter passband ripple amplitudes < 0.5 dB, stopband attenuation > 30 dB). In view of the long recording time across minimally 5 daily sessions, we refrained from extracting single-unit spike waveforms. To maximize comparability between sessions, we measured the departure of evoked multiunit spike rates from a predefined prestimulus firing rate. To this end, we adjusted the threshold to extract spikes, individually for each session, to yield a spike rate of 30 Hz from spontaneous activity. All evoked spike rates in this study are presented as the difference between evoked spike rates observed in the trial epochs and the prestimulus spontaneous level.

### Quantification

Experimental control and analysis was conducted using Simulink/Matlab (MathWorks, behavioral training: R2014b, analysis: R2020a). Intrinsic imaging was done using HelioScan (Langer et al., 2013). Measures of effect size were calculated as the area under the receiver operating characteristic curve (AUC). AUC = 0.5 signifies random performance, whereas AUC values of zero and one signify perfect separation. Statistical testing was done using the nonparametric statistical inference tests sign test and the Mann–Whitney *U* test.

## Results

Mice were trained on TTEBC for at least five sessions (60 trials per session) as previously done (Joachimsthaler et al., 2015; Fig. 1*A*). Briefly, a single-whisker deflection of whisker E1 lasting 250 ms (15 periods of a 60 Hz sinusoidal whisker deflection with 5° amplitude) served as CS. The ensuing stimulus-free memory period (Trace) lasted another 250 ms before the US, an air puff against the cornea, was delivered. Learned behavior, CR, consisted of the closure of the eye during CS presentation (Fig. 1*A*).

### Spatial generalization of behavior

Plastic changes on the level of the map as well as the columnar network were shown to be specific to the barrel column receiving the CS signals (Galvez et al., 2006; Joachimsthaler et al., 2015; but see Harris et al., 1999, for a different whisker-based performance). To confirm the columnar specificity with behavioral means, we tested the ability of four mice trained on a single-whisker CS to behaviorally generalize to other whiskers. After conditioning a stimulus at whisker E1, we presented 50 paired trials using E1 as CS and 10 randomly interspersed, unpaired trials (no US) using test whisker stimuli, which had never been presented before to the animal. One adjacent (neighbors with common border; D1 in two mice and δ in two others), one near (one intercalated whisker, C1), and one far whisker (three intercalated whiskers, α) were tested this way (Fig. 1*B*). The first post-learning session started with the far whisker, followed by the near whisker, and the adjacent whisker was tested in the last session. Eyelid trajectories (Fig. 1*C*, top, representative examples from one mouse) revealed that adjacent whisker stimuli readily evoked a CR that was similar in its timing and extent to the one generated by stimulation of the trained whisker. However, generalization was clearly limited to the first-order neighbors. With near and far stimuli, sometimes initial slow eyelid movement was observed, but always this was quickly aborted and even gave rise to reopening of the eye after the stimulus (Fig. 1*C*). The population responses plotted in Figure 1*D* show that neither near nor far whisker stimulation gave rise to a fully formed CR (lid position difference, all in millimeters, at the end of the trace period; test minus trained whisker, adjacent whisker, median = 0.05; iqr = 0.55; sign test, *p* = 0.39; near whisker, median = −0.72; iqr = 0.52; sign test, *p* < 0.01; far whisker, median = −0.69; iqr = 0.76; sign test, *p* < 0.01 [iqr, interquartile range]). With these findings, we demonstrate that the generalization of learned responses on the map of whiskers and respective spread of plasticity found within the cortical barrel map closely coincide (Galvez et al., 2006; Joachimsthaler et al., 2015).

### Learning-related changes in barrel column activity

**Figure 4.**
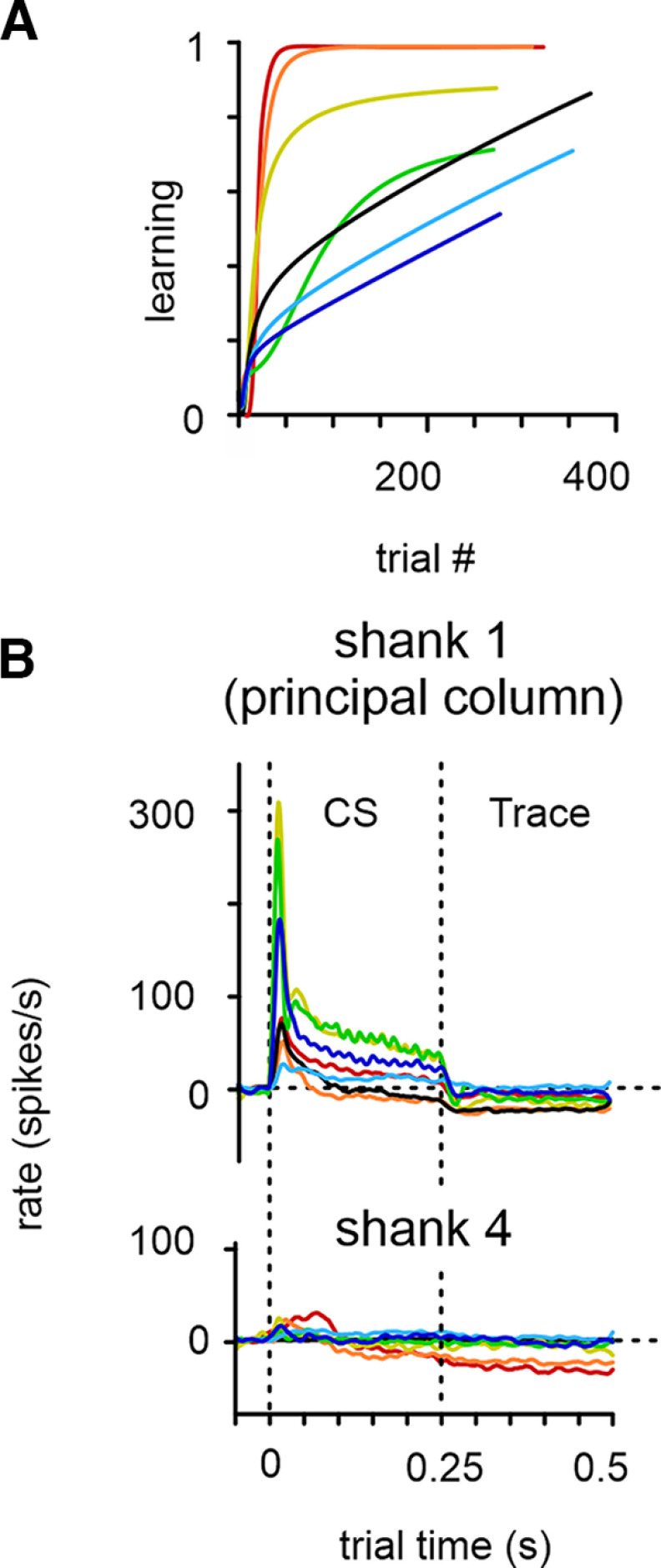
Learning-related firing suppression in the Trace interval. ***A***, Smooth polynomial fit of learning curves (linear abscissa). Each color represents one mouse (*n* = 7). ***B***, Firing rate in shank 1 and shank 4 averaged across all trials in the seven mice. Note the consistent firing rate suppression in the Trace interval, which extends to shank 4 (600 µm lateral to shank 1 located in the principal column). Color code for mice is consistent through Figures 4-6.

**Figure 5.**
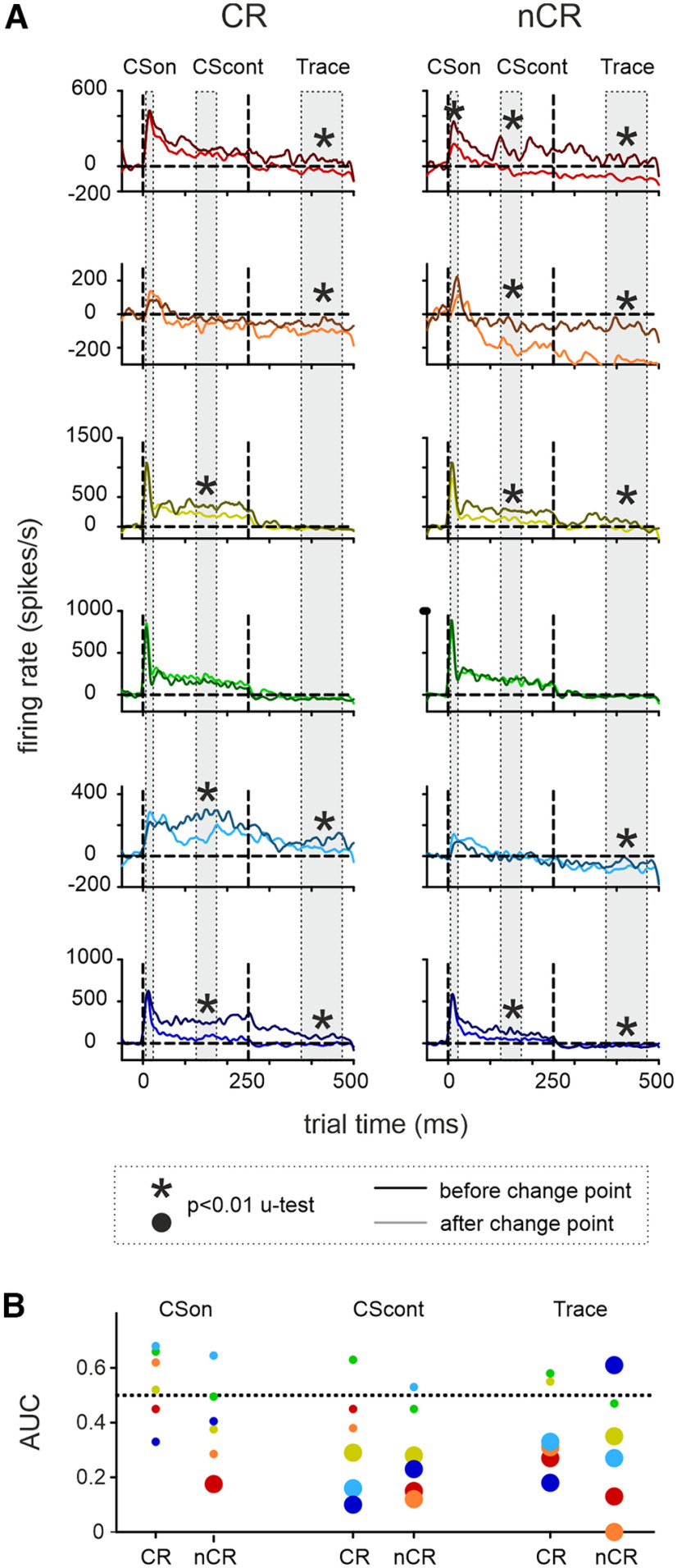
Spike rates before and after the change point. ***A***, Spike rates averaged from 40 trials before and after learning (less if change point is close to start and end of the trials series). Average firing rates obtained from electrodes located between L4 and L5/6. Dark curves depict the spike rate before the change point, light curves depict the spike rate after the change point. The CS presentation and the stimulus-free trace interval are delineated by broken lines. The gray fields indicate the analyzed epochs CSon, CScont, and Trace. Asterisk denotes significantly different rates (Mann–Whitney *U* test, *p* < 0.01, *n* = 21, 51, 101 in the three intervals). ***B***, Effect sizes (AUC) calculated from the same data (large circles are statistically significant comparisons in the *U* test). AUC signals zero effect size at a value of 0.5 (dotted line). AUC values <0.5 indicate firing rate suppression. Color code for mice is consistent through Figures 4-6.

A second group of seven mice was trained on the acquisition of TTEBC while we recorded multielectrode spike activity in the barrel cortex. Throughout Figures 2-5, these mice are labeled consistently using colors (red, orange, yellow, green, black, sky blue, and ultramarine = r, o, y, g, b, sb, um). Sorting out nonvalid trials, we obtained (323, 311, 273, 270, 373, 354, 277) valid trials. After classification into trials with CR and nCR, we were left with [(242, 81), (257, 54), (124, 149), (90, 180), (128, 245), (80, 274), (53, 224)] trials. The maximum learning score achieved varied widely among the mice ([0.99, 0.99, 0.87, 0.69, 0.85, 0.69, 0.51]).

**Figure 6.**
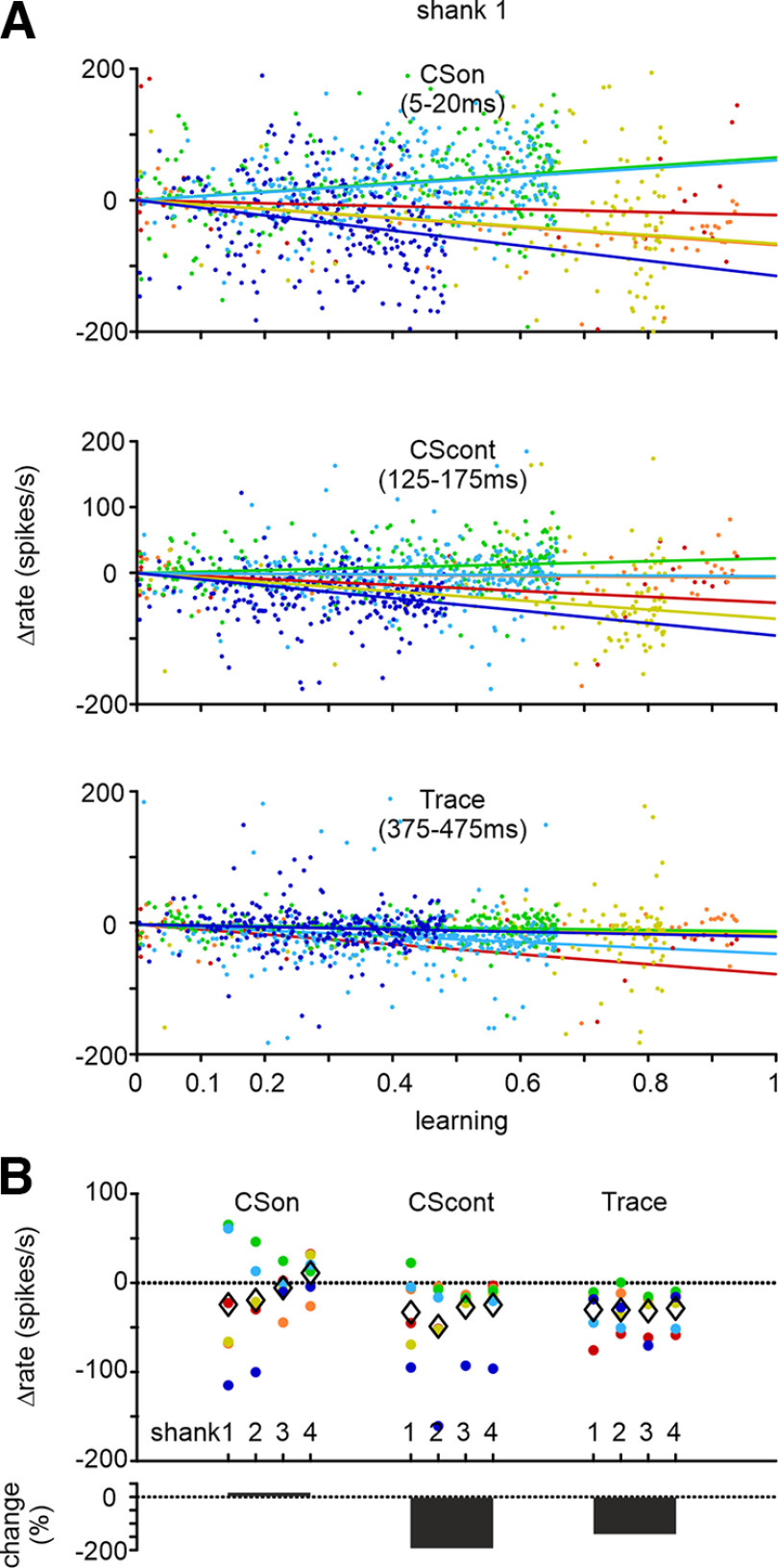
Spike rate changes (Δrate) across learning. ***A***, Firing rates averaged from electrodes located between L4 and L5/6 of shank 1, and for the three epochs CSon, CScont, and Trace, plotted across learning score (as defined by the polynomial fit to learning data; compare Fig. 2*B*). Each dot is the firing rate measured in one trial. Linear regression of this data for each mouse is shown as well. (For better visualization of the slopes, lines are shifted slightly to start all at 0 Δrate.) ***B***, Slopes of regression lines for all mice, all shanks, and all epochs (dots; mean slope, diamond). The slopes can be interpreted as the firing rate change expected if a mouse learns fully (from score 0 to 1). In CSon firing rate changes are scattered around zero. In CScont and Trace all mice show suppressed firing rates on all shanks (except 1 data point). The lower plot shows relative changes in percentage (can be >100% suppression if values approach 0). Color code for mice is consistent through Figures 4-6.

The raw binary data (nCR = 0, CR = 1), as well as a learning score obtained by a nine-trial running average of each mouse are shown in Figure 2*A*. As expected from previous work (Gallistel et al., 2004), individual learning curves rarely proceed incrementally but instead showed abrupt changes or jumps. Furthermore, the sessionwise nature of data acquisition, which is unavoidable, can affect the progression of learning scores because of consolidation of learned content during sleep between sessions (Rasch and Born, 2013) and because of uncontrolled states of the animal, which act as sources of noise (wakefulness, satiation, etc.). Our general approach took great care to minimize the latter influences by providing a thorough habituation to head fixation by running the recording session on subsequent days without gaps, having the same experimenter and laboratory context every day, and conducting the experiments always at the same time of the day. Nevertheless, the learning progress could not be fit in satisfying ways with classic S-shaped functions (e.g., the logistic function). We, therefore, resorted to two independent methods (see above, Materials and Methods). The first approach took as a point of departure that learning is monotonically increasing. It estimated, based on a monotonically increasing upper bound of the learning score, a smooth progression of the learning score by a polynomial fit of higher order (Fig. 2*B*, curves in colors). The second approach dropped all assumptions about the shape of the learning curve and estimated change points of learning scores in similar ways as done previously (Gallistel et al., 2004). Figure 2*C* shows the CUSUM (http://www.variation.com/cpa/tech/changepoint.html; Durstewitz et al., 2010; Chakrabarti et al., 2022; see above, Materials and Methods), which is the progression of the cumulative distance between the series of learning scores and their mean. The negative peaks between two points at which the CUSUM curve departed from the bootstrapped population allowed us to identify significant change points. In all mice the first such peak was accepted as the change point of learning. Cross-validating the two methods indicated an acceptable match of hallmarks of learning progression. Change points in all mice were located near the steepest slope of the smooth learning curve (Fig. 2*B*). We used the extracted change points to select trials before and after learning in Figure 5. The smooth learning curve served to plot neuronal data across learning scores in Figure 6.

To register neuronal activity in the barrel cortex during acquisition of TTEBC we used four-shank silicon probes, with eight electrodes each, in six mice. One shank was placed in the center of the principal barrel column (E1) so that the grid of electrodes would cover the full depth of gray matter vertically and as well as one or two adjacent neighboring barrel columns horizontally (Fig. 3*A*). Recordings were obtained during a minimum of five sessions (presenting 60 trials each) in which the mice were trained on TTEBC. Occasionally the most superficial electrode did not enter the cortex; however, this never happened in the principal column. Furthermore, in the sky blue mouse, recordings on shank 3 had to be discarded because of the strong electrical artifacts introduced by the galvo motor moving the vibrissa. All other electrodes gave consistent multiunit spike and LFP responses as shown in Figure 3. In the seventh mouse (black) a single shank electrode array with 16 electrodes was implanted. This array developed a short circuit that electrically connected all the electrodes. The data from this mouse showed a typical response pattern in CS and Trace epochs (Fig. 4) but did not allow us to locate the source across cortical depth and horizontal spread. We, therefore, excluded these data from the spatially defined analyses presented in Figures 5 and 6.

LFP traces (Fig. 3*B*, example from the yellow mouse) typically showed a short-latency (∼5–7 ms) negative deflection because of the first volley of the sensory signal, the amplitude of which showed two local maxima across cortical layers, one in layer L4 and another one at the border of L5/6 (abbreviation L plus number indicates the layer of that number throughout the text). The LFP sign reversed in the supragranular layers (likely L2), as reported before (Jones et al., 2000). This is the classical sensory volley at CS onset (CSon). The CSon response decreased strongly with distance to the principal barrel and largely vanished at a distance of 600 μm. In addition, a smaller negative deflection was consistently seen during the remaining presentation time of the CS (CScont) corresponding to the adapted cortical CS response. Importantly, the stimulus-free Trace period held a consistent response, this time as a positive deflection. As shown in detail below, these late responses in both CScont and Trace (here observed in the trial-averaged LFP of the first session) already show learning-related aspects. We used the LFP response features to define the following epochs for subsequent analyses: CSon, 5–25 ms; CScont, 125–175 ms; Trace, 375–475 ms (times relative to CS onset, the epochs are shown in Fig. 3*C*).

To determine the location of the electrodes with respect to the cortical layers, the LFP data were converted to CSDs (Nicholson and Freeman, 1975; Mitzdorf, 1985), one per session, in three dimensions (depth, lateral distance, trial time; Fig. 3*C*). Cortical layers were identified by slicing the CSD maps along the depth of the cortex at lateral distance zero (i.e., along the electrodes on shaft 1; Fig. 3*C.2*). In the CSon period, the early volley of sensory response generated a strong current sink with two minima, which are known to be located at L4 and L5/6 (Fig. 3*C.2*; Swadlow et al., 2002). These locations were consistently transferred to Figure 3, *B* and *D*, using black triangles. Slicing the CSD map along the surface of the cortex (across shanks), all CSD features were revealed to taper off with distance from the principal column (Fig. 3*C.1*). The double-peaked current sink during CSon (Fig. 3*C.2*) was followed by adapted activity during CScont (Fig. 3*C.4*,*C.5*) and gave rise to a double-peaked current source during Trace (Fig. 3*C.3*). The CSD data presented are averages over trials from one session. As seen below they largely reflect the results we gained using trial-resolved spike data. We did not attempt to quantitatively study trial-resolved CSD data. The reason is that CSD data, compared with spike data, show considerably higher variability from trial to trial, and effect sizes turned out to be considerably lower.

Evoked firing rate was computed as the difference in rate during stimulation and prestimulus periods (Fig. 3*D*). Confirming previous findings, the response to whisker deflection was a sharp excitatory CSon response, followed by an adapted activity during the remainder of the CS (Maravall et al., 2007; Waiblinger et al., 2015; Gerdjikov et al., 2018). In the six mice implanted with multishank electrodes, CS responses were maximal in the principal barrel column and fell off very quickly along the horizontal cortical extent. The key characteristics of spike responses—onset and adapted responses, their focus on L4 and L5/6, and the rapid horizontal decay—appear largely consistent with the spatiotemporal patterns of LFP/CSD responses (compare Fig. 3*B*,*C*).

Based on these dense multichannel electrophysiological data, our experimental strategy was to relate dynamics of psychometric data (learning scores and change points) with neurometric data (spike rates across trials). Rather than comparing large groups of learner versus nonlearner animals as has been done before (Ward et al., 2012), we strived to get at the question of how far neuronal dynamics in the individual can explain dynamics in learning behavior. To this end we exclusively focused on trial-by-trial and within-animal analyses using systematic and dense temporal and spatial sampling of neuronal data (five sessions on consecutive days, and a 4 × 8 electrode array spanning the relevant extent of cortex throughout layers and neighboring barrel columns). We show that if interindividual differences in learning speed and success are accounted for, learning behavior is dynamically reflected by neuronal signals. We refer to this dynamic relationship between learning and neuronal activity as “learning-related activity.”

Average firing rates across trial time showed a typical profile in the two main epochs, CS and Trace (Fig. 4). Data for this plot were taken from L4 to L5/6, where we obtained the strongest responses. (The black mouse showed a similar pattern, and we add it here with the cautionary note that in this animal we cannot locate the origin of firing.) From these plots it first can be observed that spike rates in shank 1 (principal column) consistently showed the classical strong, short latency excitatory CSon response. The amplitude of this response varied from animal to animal with a ranking of y-g-um-r-b-o-sb; that is, the yellow mouse yielded highest response amplitude and the sky blue one the smallest. Second, in CScont, average firing rates adapted to a lower level of firing and stayed either on a slightly elevated (five mice) or suppressed level (two mice) compared with spontaneous prestimulus firing (a finding reported before with repetitive whisker stimulation of awake rats; Gerdjikov et al., 2018). The order of spike rate amplitudes was almost identical to CSon (y-g-um-r-sb-b-o). Third, in the late Trace period spike responses were consistently suppressed, but interestingly the ranking of firing rate levels deviated rather grossly from the patterns obtained in CS. From y-g-um-r-sb-b-o in CScont, the ranking changed to the reshuffled sequence sb-um-r-g-y-b-o. These results are in line with the notion that firing rates in CS and Trace intervals are not exclusively governed by systematic correlations of firing rate amplitudes as would be expected, for example, if firing rates were transferred to the next interval by long time constants or rebound responses. The relative independence of firing in the two periods is further supported by recordings on shank 4 located in an adjacent barrel column. There CSon responses were greatly diminished, but the Trace activity appeared prominently suppressed as in the principal column.

The detected behavioral change points (compare Fig. 2) allowed us to calculate spike rates just before and after the most significant change in behavior. As before, we limited our analysis to averages taken from the most responsive zone in cortical depth between L4 and L5/6. Spike rates as calculated from 40 trials before and after the change point (Fewer trials were taken if the change point of the respective mouse occurred earlier than 40 trials) in shank 1 (principal column) were plotted together into the same graph (Fig. 5, dark color, before; light color, after). We divided the data into CR and nCR trials to judge whether the found differences could be associated with a successful motor action. We found the CSon response to be stablest across animals, whereas CScont and Trace had a clear preponderance to generate a lower firing rate after the change point. AUC effect size and the Mann—Whitney *U* test were used to compare spike counts in the time bins making up CSon (*n* = 20), CScont (*n* = 50), and Trace periods (*n* = 100) before versus after the change point. Across mice, the CSon firing rate was the stablest, whereas in the CS_cont_ and Trace periods we saw a decrement of AUC, indicating firing rate suppression as well as an accumulation of significant effects in the *U* tests (*p* < 0.01; Fig. 5*B*).

Using the smooth approximation of individual learning curves (compare Fig. 2), we next aimed to approximate the trial-to trial dynamics of the learning process reflected in spike rates and compare them across shanks (*n* = 6 mice). Figure 6*A* shows example spike rates obtained in shank 1. In the temporal domain, these data are nonsmoothed, but they are means across the electrodes located between L4 and L5/6. According to our definition, the range of learning is zero to one (i.e., zero CRs per nine trials to nine CRs per nine trials). Therefore, the average firing rate gained or lost if animals had learned the task fully well (i.e., reached learning score 1) can be estimated by the slope of the regression line calculated from the data. In CSon the regression analysis did not indicate a strong tendency of systematic spike rate changes during learning. We obtained both rising as well as decaying slopes. In CScont and even more so in Trace we systematically observed decaying slopes. In fact, only one regression line (in CScont) showed a rising slope. Figure 6*B* plots all slopes obtained on all shanks. Slopes obtained in CSon varied around zero, whereas in CScont and Trace epochs, we observed only one rising slope (of 24). In Figure 6*B*, bottom, the relative change in firing rates is shown. In the CSon epoch, learning-related activity, if it exists, is quite small in relative terms compared with the substantial relative change in CScont and Trace epochs (relative suppression can exceed 100% if the changed rate approaches zero).

### Role of barrel cortex activity in TTEBC learning

**Figure 7.**
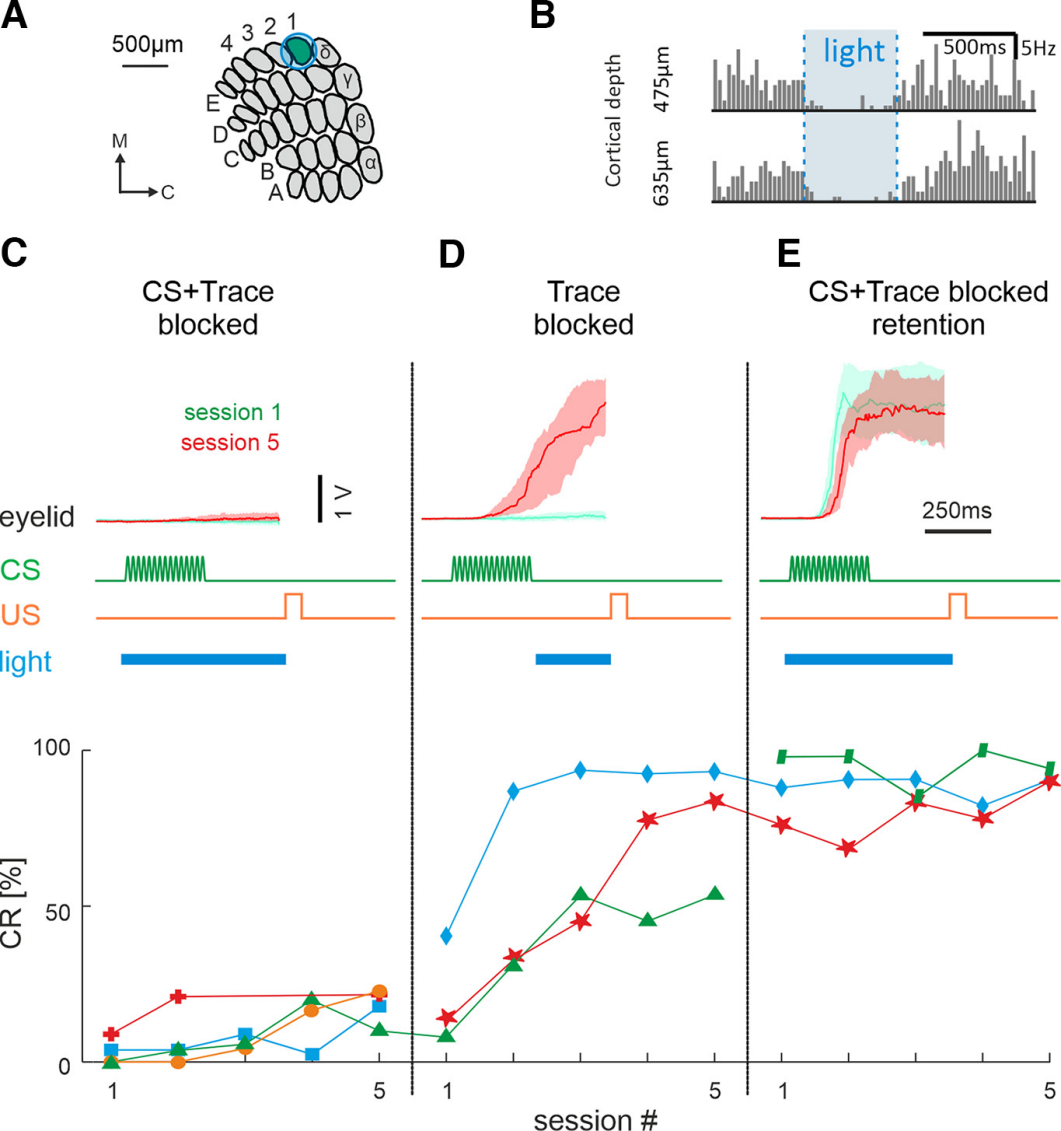
Optogenetic blockade of barrel cortex during different trial intervals (CS and Trace) and learning phases (acquisition, retention). ***A***, Schematic of barrel field with a diameter of light fiber implanted on the cortical surface. (Note that the optogenetic effect of the light applied through this fiber is more widespread than suggested by the fiber diameter.) ***B***, Effect of light application on the spiking of barrel neurons in acute preliminary experiments. Blue light activated the ChR2 expressed in cortical interneurons and blocked almost all spikes within the barrel column (VGAT mouse, method established by Guo et al., 2014). ***C***, Blockade of barrel cortex during CS and trace interval in naive mice. Top, Eyelid movements (voltage output of eyelid detector is plotted), CS, US, and light presentation during the trial are shown (light duration 500 ms). The light was not switched off abruptly but was dimmed to zero on a linear trajectory within 500 ms to avoid offset responses (data not shown; cf. Guo et al., 2014). In this experiment the learning curve of four mice was studied (bottom, different symbols and colors). ***D***, Blockade during the Trace period in naive mice (and in one mouse that was continued from experiment in ***C***, bottom, green triangles). Conventions are as in ***C***. ***E***, Blockade in CS and trace (as in ***C***) but now in trained animals testing retention. Two mice (red star, blue diamond) were continued from experiment ***D***.

We next aimed to test which learning-related changes in neuronal activity were involved in learning the sensorimotor association. To this end we used the VGAT mouse (Zhao et al., 2011; Guo et al., 2014), which allows blocking cortical circuits by activating local inhibition through activation of ChR2 expressed in GABAergic interneurons. The effectiveness of the blockade was assessed in a preliminary experiment in anesthetized animals, where the blue light, which shone through the same fiber as used later for implantation, almost totally inhibited spikes through the depth of the barrel column (Fig. 7*B*). The effect was centered on the barrel column of interest but presumably also affected other barrel columns. Importantly we used epoch-specific activation by switching the light on and off during the trial. We started training four mice on TTEBC while blocking barrel cortex in both CS and Trace epochs. None of these animals acquired the task (Fig. 7*C*) confirming previous experiments that used permanent barrel cortex lesions (Galvez et al., 2007). We then restricted the columnar blockade to the Trace epoch and found that one of the animals that did not learn in the preceding five sessions picked up in the second session and readily acquired CRs (Fig. 7*D*, green triangles). Two other naive mice, which received light in Trace only, also learned the task (Fig. 7*D*, blue diamond, red star). The two latter mice were then switched again to blockade of CS and Trace. Together with one new mouse that had learned the task before without blocking (green parallelograms), the learned performance of these animals was not affected by barrel column blockade. All seven mice used for these tests were subjected to control sessions in which the tactile CS was disabled, leaving the blue activation light (data not shown). None of the seven mice generated CRs in this control condition, showing that light alone did not assume CS function in these animals. In summary, we observed that the described learning-related activity in the barrel column during CS is critical for CR acquisition but not for retention. The described learning-related activity during Trace, on the other hand, may be related to other aspects of learning, but is not related to the acquisition of the CR.

## Discussion

We revealed three critical aspects of the role of the barrel cortex in TTEBC learning. First, we showed that single-whisker TTEBC is spatially highly specific; behavioral generalization was largely confined to the principal and adjacent whiskers but not the distant ones. Second, we revealed that both principal and adjacent barrel columns generate signals that track learning progress, independent of whether learned movements (CRs) were generated in a trial. The learning-related activity was observed late after the onset of CS, during the CScont and Trace epochs. Third, we showed that barrel cortex is only partially essential to learning implicit content (i.e., the generation of CRs): Learning-related activity in Trace is not needed for TTEBC at all, although that in CScont is only needed for task acquisition, not retention. Although the detailed function of the observed spike plasticity remains to be elucidated, we have thus opened the possibility that the observed learning-related changes may be related to learning systems storing content not directly related to the CR.

### Spatial specificity

We found a spatial limitation of single-whisker TTEBC CRs to the principal and adjacent whiskers/barrel columns. These findings neither fit the idea of strict single-whisker functional boundaries, suggested by the L4 barrel compartment (Woolsey and Van der Loos, 1970; Van Der Loos, 1976; Ma and Woolsey, 1984), nor do they match the graded and more shallow generalization performance observed with auditory TEBC in rabbits (Garcia et al., 2003). Nevertheless, our findings generally fit the literature about the extent of multiwhisker integration in the cortex (Fox et al., 2003; Higley and Contreras, 2005; Kwegyir-Afful et al., 2005; Katz et al., 2006; Urbain and Deschênes, 2007) and the subcortical tactile pathway (Simons and Carvell, 1989; Veinante and Deschênes, 1999). The location of receptive field centers and the location of neurons do not always match (Clancy et al., 2015), and multiwhisker input can generate modulatory receptive field surrounds (Estebanez et al., 2012; Ramirez et al., 2014). These interactions are reflected by dendrites and axons straddling the border between neighboring barrel columns (Kim and Ebner, 1999; Oberlaender et al., 2012). In the context of cerebellum-dependent learning as studied here, it is worth pointing out that the corticocerebellar projection system has a spatial resolution in about two barrel columns as well (Schwarz and Möck, 2001). Our findings are supported by learning-related changes in barrel size and number of dendritic spines largely confined to the principal barrel column observed previously using TTEBC (Galvez et al., 2006, 2011; Joachimsthaler et al., 2015). Finally, a generalization gradient measured from behavior in a complex sensorimotor context showed a similarly steep falloff from the trained whisker (Harris et al., 1999). Together with this prior work, our present results support the view that the basic unit contributing maximally to TTEBC includes just the principal barrel column and its next neighbors.

### Learning-related spike suppression in barrel cortex

We observed learning-related spike suppression in the barrel column during the late CScont and Trace epochs. The effect was prominent between L4 and L5/6 and is therefore consistent with the report of a significant reduction of spine density in the apical dendrites of L5 during TTEBC (Joachimsthaler et al., 2015). Our optogenetic blockade experiments showed that acquisition of the task is dependent on activity within the barrel column. Although these results are suggestive of a causal link between activity changes and structural plasticity, the existence of such a link, its direction and its mechanisms, need to be confirmed and elucidated by future research. Previous work by other authors on L5 spiking during EBC focused on the short latency response to the CS (CSon in our terminology) and reported an elevation of firing rate during TTEBC in rabbits (Ward et al., 2012). We confirm here that L5 is within the zone showing strong learning-related changes. However, extending the focus on late responses, we revealed that the bulk of learning-related changes rather occur later in CScont and Trace. We think this advance was made possible by using the highest experimental control in head-fixed animals and abolishing interindividual variability by applying within-neurons and within-animal analyses rather than comparisons of learning and control cohorts of animals as done previously.

The finding of significant learning-related changes at delays of about half a second after CS onset suggests that the learning-related effect is mediated by long-range feedback network interactions. However, there is the remaining possibility that the effects are extended in time simply by the short-term plasticity of local synapses (sensory adaptation) or membrane processes with long time constants. We think that these local phenomena do not play a dominant role to account for our observations for the following reasons. The CSon in our data does not contain consistent learning-related changes. Therefore, learning-related suppression, observed later in the trial, is unlikely to be directly mediated by the short-delay sensory volley. The observed spike rate suppression could be supported by long-lasting afterhyperpolarization, short-term synaptic suppression, or local inhibition (Chung et al., 2002; Butovas et al., 2006). Such processes, however, would be expected to relax after the excitatory CS input is terminated. However, if anything, suppression grows stronger during Trace (compare Fig. 5). Finally, if CS offset triggered some type of rebound suppression, we would expect a negative correlation between rates in CScont and Trace. However, the learning-related suppression of firing rate smoothly continues from CScont into Trace, and we observed inconsistent ranking of rates in CScont and Trace across animals. We, therefore, hold it unlikely that the learning-related activity, as observed here, is exclusively based on cellular mechanisms or local synapses, and we favor the view that barrel cortex generates the found learning-related activity using cooperative interactions within a larger network.

### Learning-related signals in the barrel cortex

Silencing the barrel cortex prevented TTEBC acquisition but not retention, an impact clearly beyond the mere interruption of the ascending sensory pathway. Further, we demonstrate that learning-related activity was independent of the presence of CRs in individual trials. In summary, within the framework of implicit learning, the barrel cortex does not hold the memory, it is not a necessary gateway for sensory information, and does not directly control eyelid movement.

Which memory networks might interchange signals with the barrel cortex during TTEBC? The major contenders are intracortical projections, thalamic innervation from the posterior medial thalamus, or neuromodulatory inputs (Aronoff et al., 2010; Eggermann et al., 2014). The medial prefrontal cortex (mPFC) generates a memory signal during Trace that is believed to be projected via the pontine nuclei to the cerebellar association centers (Siegel et al., 2012). One possibility is, therefore, that barrel cortex engages via intracortical connections in reverberatory interactions with mPFC and related areas, helping to generate these memory signals. The barrel cortex could as well interact more directly with the cerebellum, either via its own massive and highly precise projections to the pontine nuclei (Schwarz and Möck, 2001) or via the mesodiencephalic junction and farther to the inferior olive (Apps and Watson, 2013). Through these pathways the barrel cortex is able to gain access to specific mossy and climbing fibers inputs to the cerebellum conveying contextual (Bouton, 2004) and/or error signals (Mathis et al., 2017). It has been proposed that the cerebro-cerebellar loop connects cortical areas with the cerebellum in multiple closed-loop fashion (Schwarz and Thier, 1999; Strick et al., 2009; Proville et al., 2014). Although a demonstration of such cortico-cerebellar closed loops is lacking for barrel cortex, the known outline of cerebellothalamic afferent pathways may in principle allow for such an arrangement (Teune et al., 2000; Kuramoto et al., 2009; Schäfer and Hoebeek, 2018).

Although both the mentioned interactions (with other cortical areas or directly with the cerebellum) can in principle serve the formation of implicit memory, the learning-related activity present in Trace during acquisition and in CScont/Trace during retention is not explained by implicit learning; blockade of this trial period and learning phase had no effect on CR generation. This opens the possibility that barrel cortex has a role beyond implicit association for the purpose of generating a CR, perhaps to learn the rules of the game.

## Synthesis

Reviewing Editor: David Franklin, Technische Universitat Munchen Decisions are customarily a result of the Reviewing Editor and the peer reviewers coming together and discussing their recommendations until a consensus is reached. When revisions are invited, a fact-based synthesis statement explaining their decision and outlining what is needed to prepare a revision will be listed below. The following reviewer(s) agreed to reveal their identity: Joshua Brumberg, Ehsan Arabzadeh. Note: If this manuscript was transferred from JNeurosci and a decision was made to accept the manuscript without peer review, a brief statement to this effect will instead be what is listed below.

Your manuscript has been re-reviewed by the same reviewers. Both reviewers appreciated the revisions that addressed their previous comments and concerns. They both stated that this work advances the field, and they highlighted the very nice experimental paradigm and analysis techniques that allowed you to study the neuronal correlates of associative learning in the whisker barrel cortex. There are only a few small issues that I encourage you to revise, along with a point-by-point response to the reviewers.

Reviewer 1

Silva-Prieto and colleagues demonstrate that whisker eye-blink trace learning is a robust phenomenon that is largely not transferable beyond the principal whisker (slightly to adjacent barrel columns) and that via optogenetic manipulation they show that the barrel cortex plays a key role in the learned behavior (conditioned behavior).

Based on the document this is a revised version of a submission made originally to the Journal of Neuroscience the authors appear to be very responsive to the original critiques and has limited my feedback.

Pg. 10 line 230-231 - the anacronym CUSUM (cumulative sum control chart) should be defined on first use and in (see Methods and Fig 2C....) this section is in the methods so the reference is not clear - a subsection of the methods.

How consistent was the change point (trial number) between animals?

Did LFP amplitudes and latencies remain consistent over trials?

Figure 2C the utility of the gray clouds (1000 curves plotted on top of each) other is not clear.

Reviewer 2

The authors have addressed the issues raised in my earlier review of this manuscript. I find the current version suitable for publication in eNeuro.
